# Construction and validation of 3-genes hypoxia-related prognostic signature to predict the prognosis and therapeutic response of hepatocellular carcinoma patients

**DOI:** 10.1371/journal.pone.0288013

**Published:** 2023-07-05

**Authors:** Yunxun Liu, Bingbing Shen, Ting Huang, Jianguo Wang, Jianxin Jiang

**Affiliations:** 1 Department of Urology, Renmin Hospital of Wuhan University, Wuhan, Hubei, PR China; 2 Institute of Urologic Disease, Renmin Hospital of Wuhan University, Wuhan, Hubei, PR China; 3 Department of Hepatobiliary Surgery, Renmin Hospital of Wuhan University, Wuhan, Hubei, PR China; 4 Department of Anaesthesiology, Renmin Hospital of Wuhan University, Wuhan, Hubei, PR China; Tanta University Faculty of Medicine, EGYPT

## Abstract

**Background:**

Previous studies have shown that the hypoxia microenvironment significantly impacted tumor progression. However, the clinical prognostic value of hypoxia-related risk signatures and their effects on the tumor microenvironment (TME) in hepatocellular carcinoma (HCC) remains hazy. This study aimed to conduct novel hypoxia-related prognostic signatures and improve HCC prognosis and treatment.

**Methods:**

Differentially expressed hypoxia-related genes (HGs) were identified with the gene set enrichment analysis (GSEA). Univariate Cox regression was utilized to generate the tumor hypoxia-related prognostic signature, which consists of 3 HGs, based on the least absolute shrinkage and selection operator (LASSO) algorithm. Then the risk score for each patient was performed. The prognostic signature’s independent prognostic usefulness was confirmed, and systematic analyses were done on the relationships between the prognostic signature and immune cell infiltration, somatic cell mutation, medication sensitivity, and putative immunological checkpoints.

**Results:**

A prognostic risk model of four HGs (FDPS, SRM, and NDRG1) was constructed and validated in the training, testing, and validation datasets. To determine the model’s performance in patients with HCC, Kaplan–Meier curves and time-dependent receiver operating characteristic (ROC) curves analysis was implemented. According to immune infiltration analysis, the high-risk group had a significant infiltration of CD4+ T cells, M0 macrophages, and dendritic cells (DCs) than those of the low-risk subtype. In addition, the presence of TP53 mutations in the high-risk group was higher, in which LY317615, PF−562271, Pyrimethamine, and Sunitinib were more sensitive. The CD86, LAIR1, and LGALS9 expression were upregulated in the high-risk subtype.

**Conclusions:**

The hypoxia-related risk signature is a reliable predictive model for better clinical management of HCC patients and offers clinicians a holistic viewpoint when determining the diagnosis and course of HCC treatment.

## Introduction

Hepatocellular carcinoma (HCC) is one of the most malignant and aggressive liver cancers and the fourth leading cause of cancer-related mortality worldwide [1]. The 5-year survival rate should be more than 70% if identified at an early stage, with surgical resection offering a favorable prognosis [2]. However, most patients already have advanced disease when diagnosed, with China’s 5-year survival rate being only 12.5% [3]. The poor prognosis and high mortality of HCC should be attributed to the symptoms and physical characteristics of HCC that are difficult to identify. Therefore, to benefit from novel therapeutics, it is vitally necessary to identify the pertinent factors influencing the prognosis of patients with HCC.

Generation of a hypoxic environment are common features of advanced cancers due to limited blood supply [4]. It was discovered that hypoxia could promote tumor progression by promoting cell proliferation or angiogenesis and preventing cell death by provoking adaptive responses [5]. It has been found that in HCC, hypoxia accelerates the aggressiveness of HCC cells through hypoxia-induced Sonic Hedgehog signaling pathway, epithelial-to-mesenchymal transition [6], and regulating the mitochondrial activity [7].

The present work speculated that hypoxia had specific prognostic significance for HCC. Through a systematic study, new predictive features were constructed and acknowledged as an independent prognostic indicator for HCC patients. The correlations of risk with immune cell infiltration, somatic cell mutation, drug sensitivity, and potential immune checkpoints were systematically assessed based on the hypoxia-related signatures to improve HCC prognosis and treatment.

## Materials and methods

### Data sources

The discovery cohort was chosen from the TCGA LIHC dataset [8], which includes 374 HCC samples (T) and 50 nontumor liver samples (N). Transcriptome RNA-seq data (STAR-Counts), including row counts reads and Fragments Per kilobase of transcript per Million mapped reads (FPKM), simple nucleotide variation data (VarScan), and corresponding clinical follow-up information of the TCGA LIHC dataset were downloaded from the TCGA GDC (https://portal.gdc.cancer.gov/). GSE14520 (N = 241, T = 247) series [9] was selected as the validation cohort, which was based on the GPL3921 platform [HT_HG-U133A] (Affymetrix HT Human Genome U133A Array). Gene expression and clinical data of the GSE14520 were originated from the GEO database (https://www.ncbi.nlm.nih.gov/geo/). Gene expression and clinical data of 232 HCC samples in the LIRI-JP data portal [10] were originated from the ICGC database (https://dcc.icgc.org/).33 hypoxia-related human ontology gene sets were downloaded from the Molecular Signatures Database v7.5.1 (MSigDB)(http://www.gsea-msigdb.org/gsea/msigdb/index.jsp) via searching by hypoxia. The “edgeR” package was used in the two cohorts to standardize the raw data. The “sva” package was utilized to remove batch effects [11].

### Identification of significant HGs

Via the gene set enrichment analysis (GSEA, version 4.2.3), the enrichment of hypoxia-related gene sets was identified, where TCGA-LIHC was expression datasets and hypoxia-related human ontology gene sets were the database. After 1000 permutations for each analysis, a nominal (NOM) p-value of 0.05, a normalized enrichment score (NES) >1.5, and a false discovery rate (FDR) of 0.15 were used as the criteria to group the GSEA results into HCC samples and non-tumor liver samples groups. The core HGs were obtained by screening the significantly upregulated hypoxia-related gene sets in HCC samples from the GSEA via leading edge analysis. Afterward, differentially expressed HGs, based on the above hypoxia-related gene sets between HCC and normal tissues in the TCGA dataset and GSE14520 were identified with the “limma” package (adjusted p-value <0.05 and |log2FC| >0.6). Overlapping HGs ground on GSE14520 and TCGA were employed for the following analysis. A weighted gene co-expression network analysis (WGCNA) was created in the TCGA-LIHC cohort using the "WGCNA" package [12]. After cluster analysis, we constructed Pearson correlation matrices and generated weighted neighbor-joining matrices to emphasize strong correlations and penalize weak correlations. A scale-free network was constructed using the function powerEstimate soft threshold to select the best soft threshold power β = 12 (fit value R2 to 0.85). Based on work of Xu et al. [13], a topological adjacency matrix (TOM) was generated. The TOM-based correlation dissimilarity measure was set at a minimum number of genes/modules of 25, resulting in the generation of 2 modules. Next, we calculated the correlation and significance of the clinical traits and each module after importing the clinical data from the TCGA-LIHC sample and matching the expression sample. The module with the highest absolute module significance contained the hub HGs.

### Development and validation of the hypoxia-related prognostic signature for HCC

10 HGs associated with HCC prognosis were analyzed using univariate Cox regression (p < 0.05). LASSO Cox regression adds a regular term based on general linear regression, which was performed to eliminate false-positive prognostic-associated HGs. HCC patients in the TCGA LIHC dataset was randomly been divided into training cohort (n = 186) and testing cohort (n = 184), the GSE14520 dataset (n = 221), the LIRI-JP dataset (n = 222) and the whole TCGA LIHC dataset (n = 370) were selected as the validation cohorts. A risk signature was established by including individual normalized gene expression values weighted based on the training cohort by their LASSO Cox coefficients as follows: (FDPS*0.241806768) + (SRM*0.559394902) + (NDRG1*0.229623136), and patients were classified into high-/low-risk subtypes stratified by the median risk score in the training cohort. In order to assess the differences in patient survival between the high-risk and low-risk subgroups, Kaplan-Meier analyses were performed. The predicted sensitivity and specificity of the hypoxia-related risk model based on training, testing, and validation cohorts were further confirmed using the time-dependent receiver operating characteristic (ROC) curves and matching areas under the curve (AUC) values. Univariate and multivariate Cox regression analyses were conducted to verify the hypoxia-related signature’s prognostic utility. A nomogram and calibration plots were created using the "rms" R program to provide HCC patients with a prediction that included clinicopathological factors. Stratification survival analyses were created to investigate the connection between risk score, overall survival (OS), and clinical traits of HCC patients.

### Associations of the prognostic signature with TME, single-nucleotide polymorphisms, drug sensitivity, and immune checkpoints

The infiltration levels of 22 types of immune cells of HCC sample ground on GSE14520 and TCGA were calculated based on the R package“CIBERSORT” [14]. To estimate the score of immune infiltrating cells and the activity of the pathway, single-sample gene set enrichment analysis (ssGSEA) and GSEA (NOM p-val<0.05, NES>1.6, FDR qval<0.06) were performed. Tumor Immune Single-cell Hub (TISCH) [15] is a scRNA-seq database focusing on TME. In this work, we used datasets derived from TISCH to decipher the TME of HCC patients at single cell level. The R package "maftools" performed single-nucleotide polymorphism (SNP) analysis to investigate the gene mutation profile of the risk signature. Using the R package "pRRophetic" [16], the half-maximal inhibitory concentration (IC50) between the high- and low-risk groupings were calculated, and the Wilcoxon test was performed to evaluate the IC50 for the subgroups. After log2 conversion, the expression level of potential immunological checkpoints was determined.

### Statistical analysis

R (version 4.2.0) and the associated packages were used for all computational and statistical studies. In addition, the R package “pRRophetic” was finished in R (version 4.0.0). Two-tailed p values < 0.05 were considered statistically significant.

The Wilcoxon test was used to compare the risk score across various clinical follow-up subtypes and the levels of immune cell infiltration, drug sensitivity, and immune checkpoint expression between the high and low subtypes in the TCGA and GSE14520 cohorts.

## Results

### Identification and extraction of differentially expressed HGs in HCC

**Fig 1:**
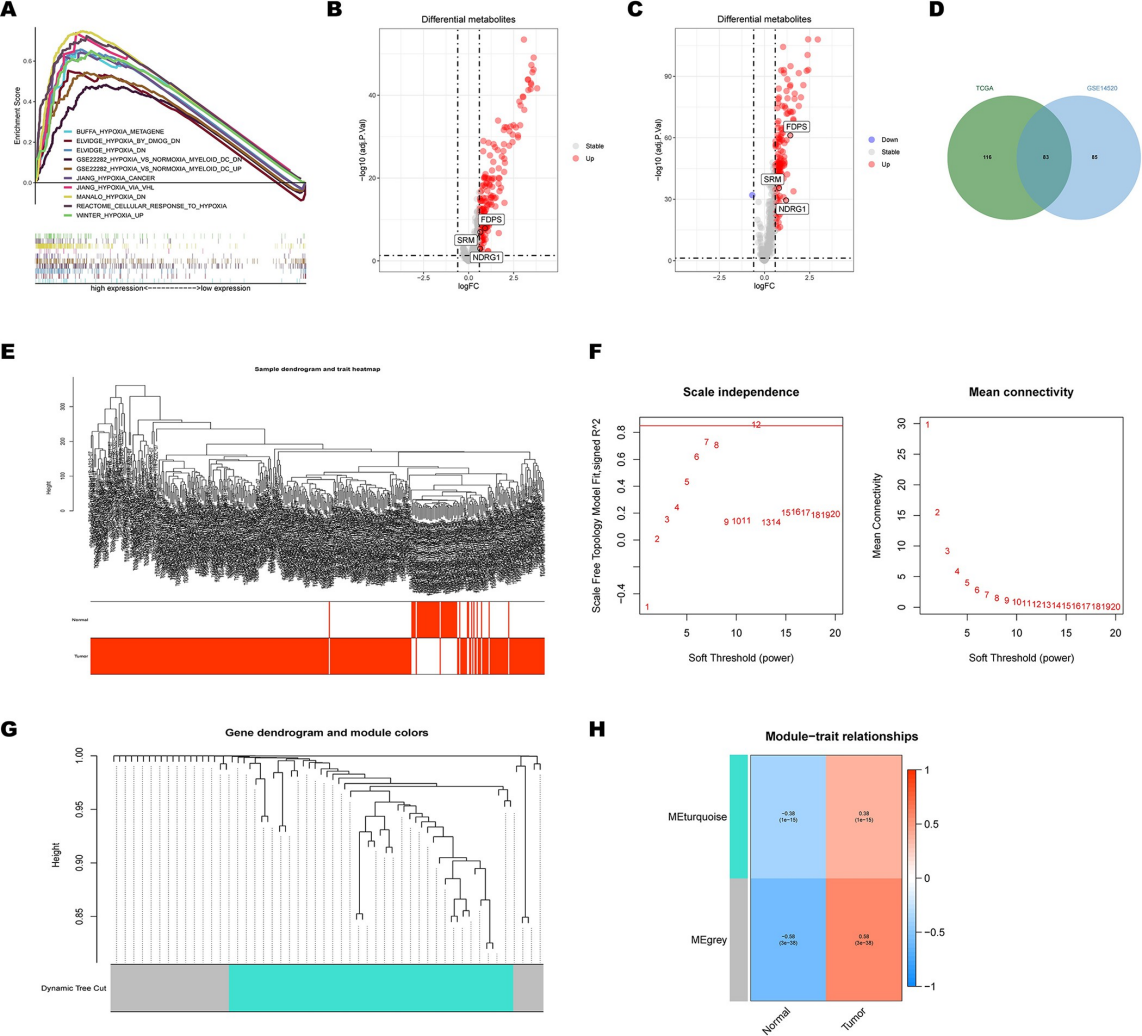
Extraction and WGCNA of differentially expressed hypoxia-related genes (HGs). (A) The multiple GSEA for 10 hypoxia-related gene sets are based on the TCGA datasets. (B) The volcano plot for the 199 HGs in the TCGA datasets. (C) The volcano plot for the 168 HGs in the GSE14520 datasets. (D) The identification of the 83 overlapping HGs ground on GSE14520 and TCGA. (E) Sample dendrogram and trait heatmap for the detection of outliers ground on the TCGA datasets. (F) Scale-free topology model fit (left) and mean connectivity (right) for the appropriate soft threshold power. The power selected was 12. (G) Clustering dendrograms of the co-expression network modules of HGs. (H) Module‑trait relationships between the tumor traits and module, correlation coefficient, and p-values are shown.

The RNA sequencing data and clinical follow-up data of 374 HCC and 50 non-tumor samples were obtained from TCGA portal. Meanwhile, we performed GSEA based on 33 human ontology gene sets related to hypoxia obtained from MSigDB. Among them, GSEA showed that 10 gene sets, including: “BUFFA_HYPOXIA_METAGENE“,“ELVIDGE_HYPOXIA_BY_DMOG_DN”, “ELVIDGE_HYPOXIA_DN”,”GSE22282_HYPOXIA_VS_NORMOXIA_MYELOID_DC_DN”,”GSE22282_HYPOXIA_VS_NORMOXIA_MYELOID_DC_UP”,”JIANG_HYPOXIA_CANCER”,”JIANG_HYPOXIA_VIA_VHL”,”MANALO_HYPOXIA_DN”,”REACTOME_CELLULAR_RESPONSE_TO_HYPOXIA”and”WINTER_HYPOXIA_UP” were significantly enriched in HCC samples (Fig 1A). A total of 647 core enrichment genes were discovered to be HGs through leading-edge analysis of the aforementioned 10 gene sets. For recognition of HGs in tumor samples, the cutoff log2FC was set in 0.6,83 HGs (Fig 1D), which were significantly upregulated in both TCGA (Fig 1B) and gse14520 (Fig 1C). After that, 83 HGs were set to WGCNA to evaluate the gene expression matrix of 374 HCC samples in the TCGA cohort. Additionally, the hclust function was used to draw the cluster dendrogram from the expression matrix calculated using the mean value method. To exclude samples that are clearly outliers, a truncation value of 500 was chosen for the height of the sample’s clustering tree (Fig 1E). The soft threshold power was set to 1–20 to ensure high-scale independence (R2 = 0.85) and low mean connectivity (near 0) when β = 12 (Fig 1F), in which only two modules were retained (Fig 1G). Fig 1H displays the module-trait connections that the Pearson correlation test revealed. The grey module related to tumor samples with 24 HGs had the deepest color (r = 0.58, p < 0.0001), regarded as the crucial module for further analyses.

### Development and validation of the hypoxia-related prognostic signature for HCC

**Fig 2:**
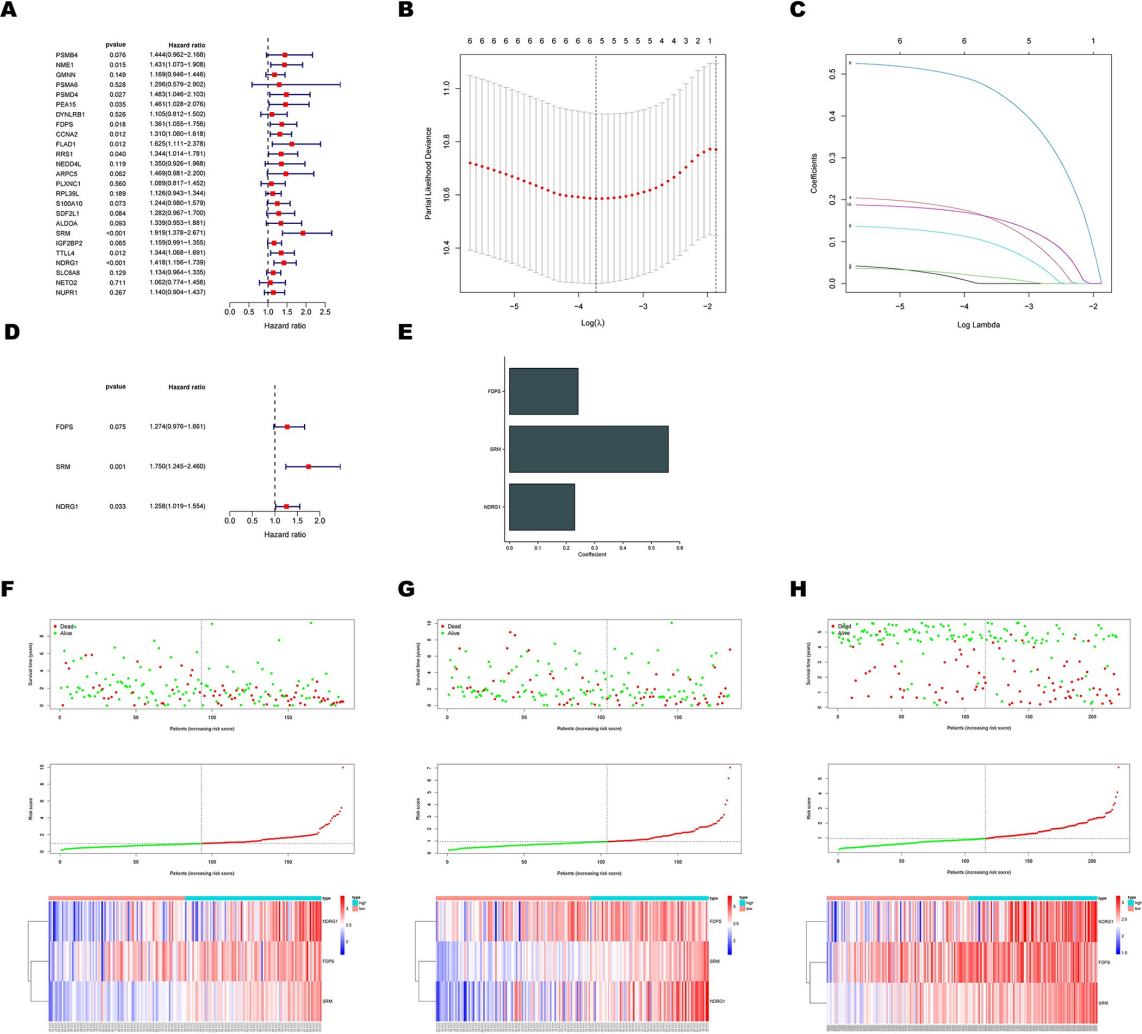
Construction and validation of prognostic risk signature of HGs for HCC patients. (A) Forest plot of univariate Cox regression analyses for 25 HGs related to overall survival ground on the TCGA dataset. (B) The partial likelihood deviance plot. (C) The Lasso regression coefficient profiles. (D) Multivariate Cox regression analyses of 3 core HGs. (E) Cox coefficients distribution of the three core HGs.(F-H) Survival status of patients, risk plot distribution, and heatmap of expression of 3 core HGs in the (F) training, (G) testing, and (H) GSE14520 cohorts.

**Fig 3:**
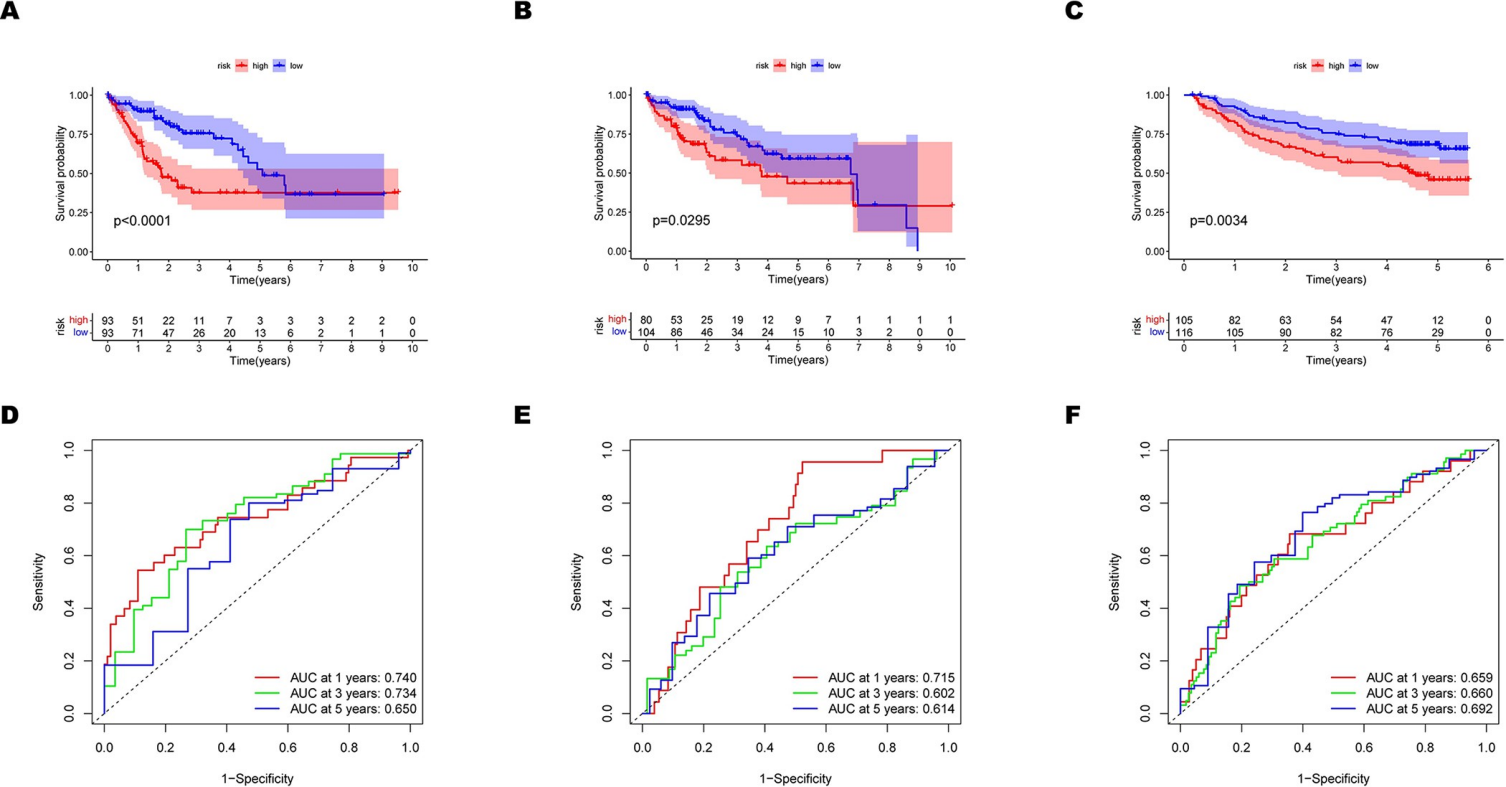
Evaluation of predictive power of the selected 3 HGs signature. (A-C) Kaplan–Meier survival curves for the risk signature based on the training, testing and GSE14520 cohort. (A) 186 cases in the training cohort. (B) 184 cases in the testing cohort. (C) 221 cases in the GSE14520 cohort. (D-F) Receiver operating characteristic (ROC) curves for the risk signature in the training, testing and GSE14520 cohort. (D) training cohort. (E) testing cohort. (F) GSE14520 cohort.

After univariate Cox regression analysis (Fig 2A), the prognostic value of 10 HGs remarkably related to the OS of HCC patients (p < 0.05) was explored in the whole TCGA cohort(n = 370). Afterward, the LASSO regression further eliminated false-positive prognosis-associated HGs, and a 3-gene signature was eventually established (Fig 2B, 2C). SRM(p = 0.001) and NDRG1(p = 0.033) were considered independent prognosis genes (Fig 2D), after Multivariate Cox regression further screening. The risk score formula was as follows (Fig 2E): (FDPS*0.241806768) + (SRM*0.559394902) + (NDRG1*0.229623136). Fig 2F–2H and S1A-S1C Fig described the risk survival status and scores of HCC patients whose risk score was calculated according to this formula in training (n = 186), testing(n = 184), and validation cohorts (GSE14520, n = 221; LIRI-JP, n = 232). The training cohort’s median risk score was used to categorize individuals into high-and low-risk subgroups. The heatmap results demonstrated that all HCC patients had higher expression of FDPS, SRM, and NDRG1 as risk factors as their risk scores increased. The Hypoxia-Related Signature may have prognostic significance, as shown by the Kaplan-Meier survival analysis (training cohort: p < 0.001; testing cohort: p = 0.0295; GSE14520: p = 0.0034, LIRI-JP: p = 0.0045, Fig 3A–3C, S1D Fig), which revealed that patients at high risk had a lower chance of survival than patients at low risk. Moreover, the prediction sensitivity and specificity of the Hypoxia-Related Signature were evaluated using a time-based receiver operating characteristic (ROC) curve. The 1-, 3-, and 5-year AUC values of ROC curves for the Hypoxia-Related Signature in the training cohort were 0.740, 0.734, and 0.650, respectively (Fig 3D). The 1-, 3-, and 5-year AUC values of ROC curves in the testing cohort were 0.715, 0.602, and 0.614 (Fig 3E). And the corresponding 1-, 3-, and 5-year AUC values of ROC curves in the GSE14520 cohort were 0.659, 0.660, and 0.692 (Fig 3F). In the LIRI-JP cohort, the corresponding 1-, 3-, and 4-year AUC values of ROC curves were 0.792,0.797 and 0.717 (S1E Fig). As a result, the accuracy of the Hypoxia-Related Signature in forecasting the prognosis of HCC was revealed.

### Correlation between the hypoxia-related signature and clinical features of patients with HCC

**Fig 4:**
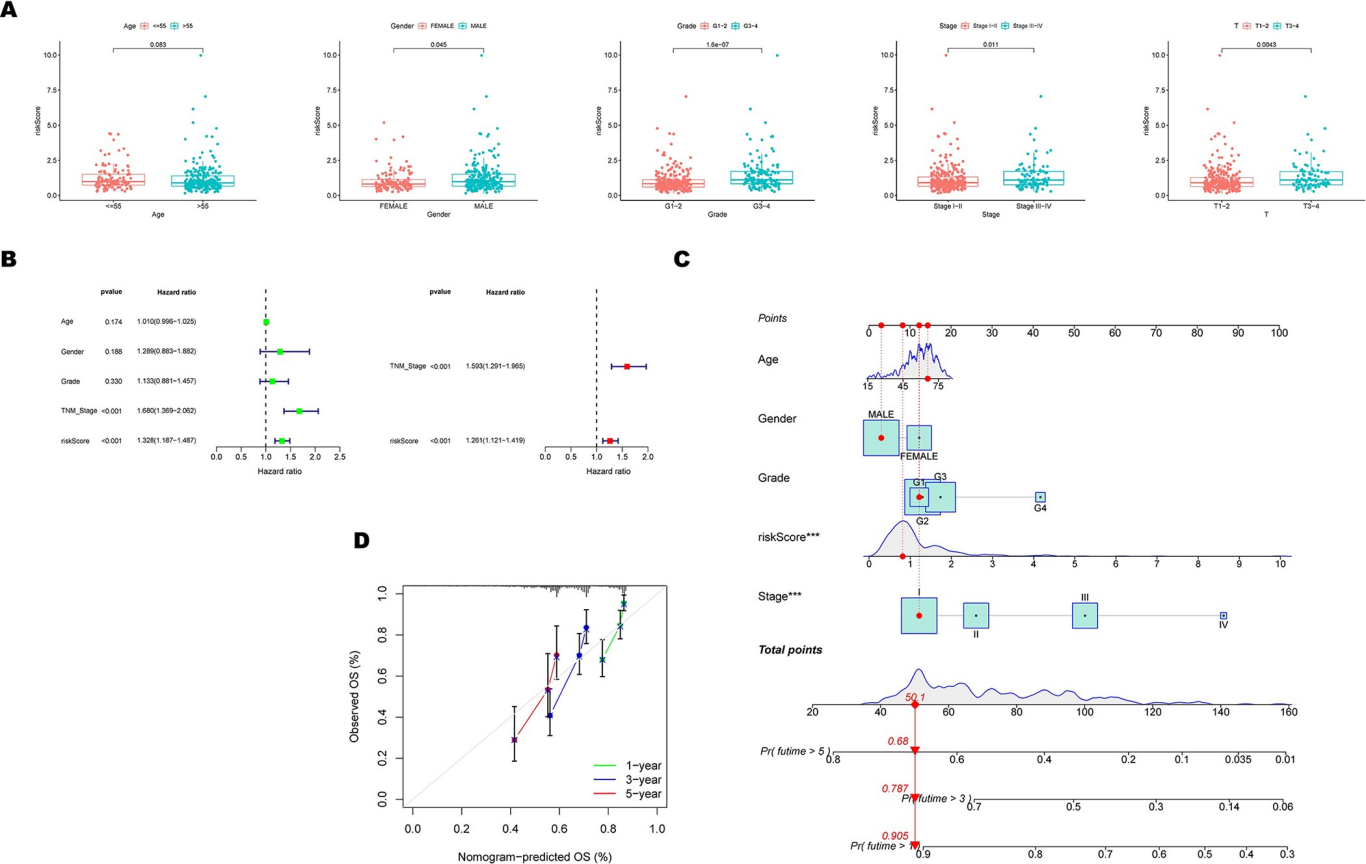
Identification of the risk signature with the overall survival and clinicopathological characteristics of HCC patients ground on the TCGA dataset. (A) Relationships between the risk score and clinicopathological characteristics of HCC patients. (B) Forest maps of the univariate and multivariate Cox regression analysis between the risk score and clinical characteristics. (C) Nomogram predicting the survival rate at 1, 3, 5 years for HCC patients, *p < 0.05, **p < 0.01, and ***p < 0.001. (D) Calibration plots for the nomogram.

The Wilcoxon test was used to compare the differences in risk score between subgroups sorted by age, gender, tumor grade, pathological TNM stage, and pathological T stage to investigate the relationships between the hypoxia-related signature and clinical characteristics of HCC patients in the TCGA cohort. It turned to that risk scores were significantly correlated with gender (p < 0.05), tumor grade (p < 0.01), pathological TNM stage (p < 0.05), and pathological T stage (p < 0.01), whereas they showed no relationship with age (Fig 4A). After univariate and multivariate Cox regression analyses (Fig 4B), pathological TNM stage (p < 0.001) and risk score (p < 0.001) were identified as independent prognostic factors. Based on the relevant clinical feature, a predictive nomogram was created to predict the 1-, 3-, and 5-year survival rates of HCC cases (Fig 4C). The predicted and observed data were well-aligned on calibration plots (Fig 4D).

On the other hand, the same analyses were performed based on the GSE14520 cohort. Risk scores were significantly correlated with the CLIP stage (p < 0.01), BCLC stage (p < 0.01) and TNM stage (p < 0.01), whereas they showed no relationship with age and gender (S2A Fig). As univariate and multivariate Cox regression analyses (S2B Fig) showed, TNM stage (p < 0.05), BCLC stage (p < 0.05) and risk score (p < 0.01) were identified as independent prognostic factors. A predictive nomogram was built to predict 1-, 3-, 5-year survival rates of HCC cases based on the above clinical factors (S2C Fig). Meantime, the calibration curve was created to assess the consistency between the genuine value and the OS anticipated value (S2D Fig). On the LIRI-JP cohort, risk score was only significantly correlated with TNM stage (p < 0.01) (S3A Fig). TNM stage (p < 0.01) and risk score (p < 0.01) were identified as independent prognostic factors according to univariate and multivariate Cox regression analyses (S3B Fig). Based on the relevant clinical feature, a predictive nomogram was created to predict the 1-, 3-, and 4-year survival rates of HCC cases in LIRI-JP cohort (S3C Fig). The predicted and observed data were well-aligned on calibration plots (S3D Fig).

**Fig 5:**
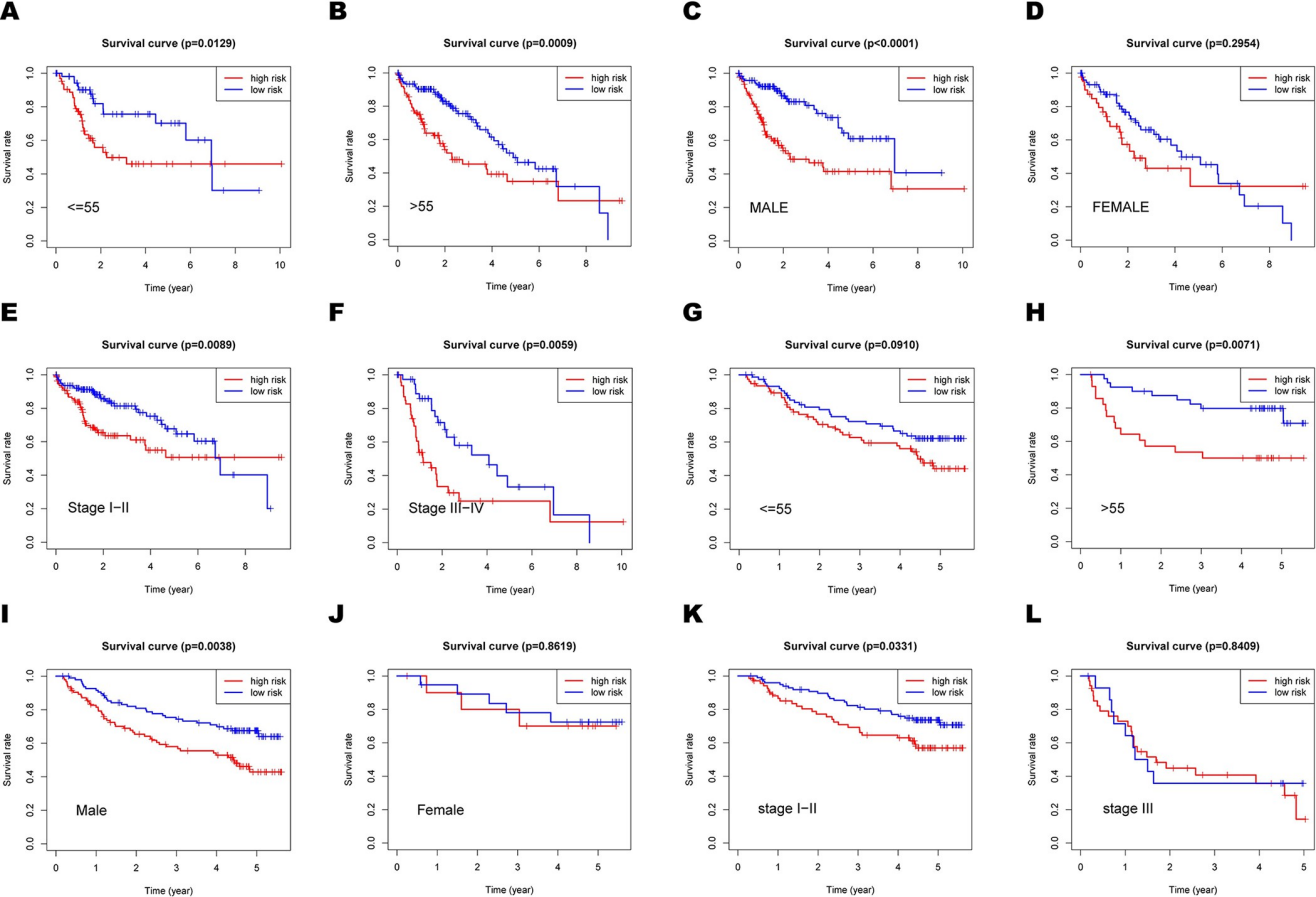
The prognostic ability of the 3 HGs signature for overall survival in multiple HCC subtypes. Kaplan–Meier curves for OS prediction in HCC subtypes of (A) age < = 55, (B) age >55, (C) Men, (D) Women, (E) Stages I–II, (F) Stages III–IV in the TCGA cohort, (G) age < = 55, (H) age >55, (I) Men, (J) Women, (K) Stages I–II, (L) Stages III in the GSE14520 cohort.

Previous studies of molecular biology have shown that stratification survival analyses could delegate predictive value of the prognostic signature [17, 18]. The three HGs signature for prognosis in numerous HCC common subtypes in the TCGA and GSE14520 cohort, which were categorized by age (55 years and >55 years), gender (Male and Female), and pathological TNM stage (Stages I–II and Stages III–IV or Stages I–II and Stages III), were also assessed using stratification survival analyses. In HCC patients older than 55, who were male, and had Stage I or II, the high-risk group had significantly worse overall survival (OS) than the low-risk group (all p<0.05; Fig 5B, 5C, 5E, 5H, 5I and 5K).

### Association of tumor immune cell infiltration with the hypoxia-related prognostic signature

**Fig 6:**
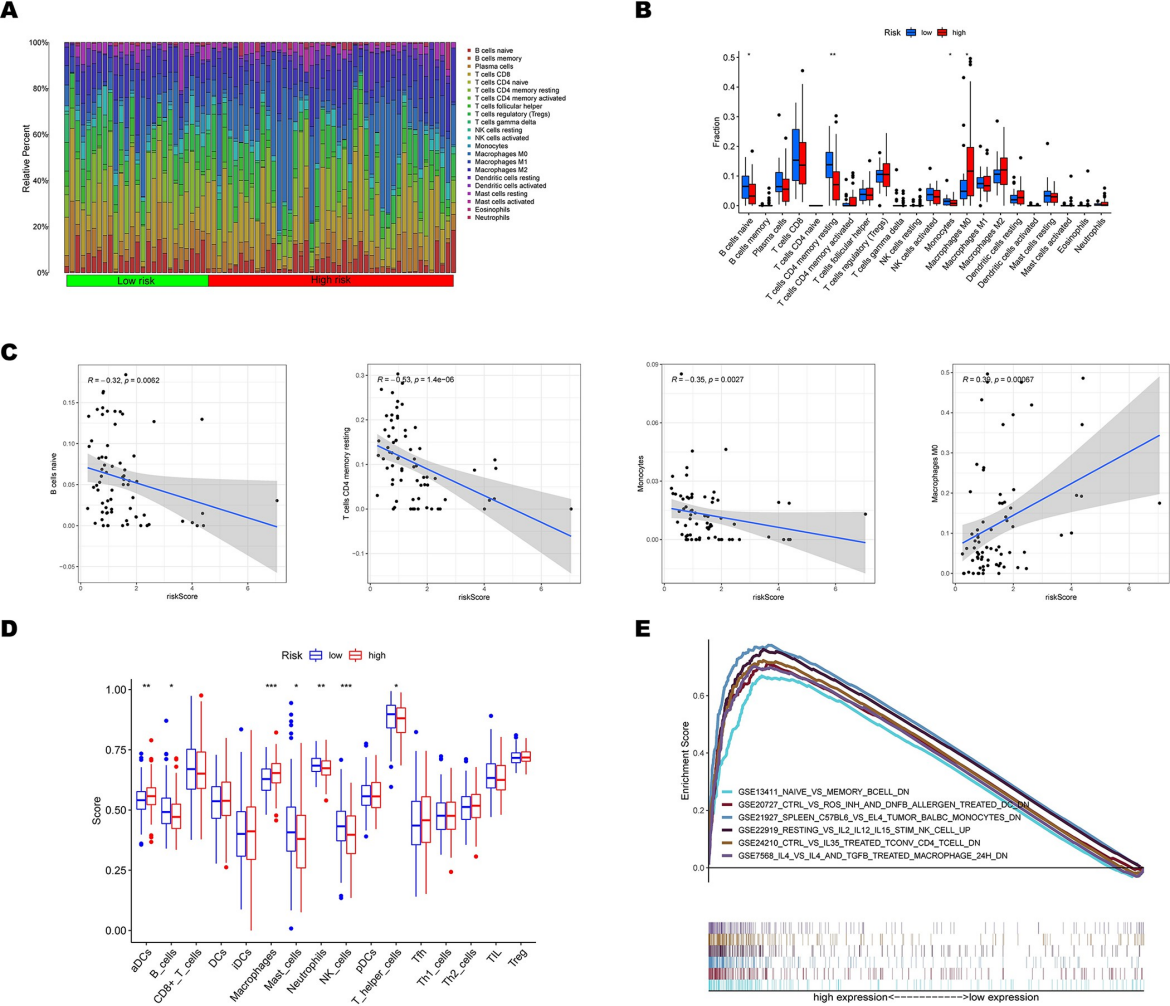
Association of tumor immune cell infiltration with the risk score in the TCGA cohort. (A) The bar graph of relative proportions of the 22 immune cells between the high and low-risk subgroups. (B) The bar graph of difference in composition of the 22 types of immune cells between two risk subgroups, *p < 0.05, **p < 0.01, and ***p < 0.001. (C) The correlation between the risk score and naive B cells, resting memory CD4 T cells, monocytes, and M0 macrophages. (D) The bar graph of the difference in the enrichment scores of 16 types of immune cells between two risk subgroups (E) The multiple GSEA for significant immune pathways based on the TCGA dataset.

The proportion of immune subsets that infiltrate tumors was analyzed using the CIBERSORT algorithm, and 22 different immune cell profiles between the high and low-risk subgroups in TCGA HCC samples were created (Fig 6A). This allowed researchers to further investigate the differences between the three genes signature and the immune microenvironment. The M0 macrophages, M2 macrophages, memory CD4+ T cells, and CD8+ T cells accounted for the highest proportion. The difference analyses showed that HCC patients of the high-risk subtype showed a lower infiltration level of naïve B cells, resting CD4+ memory T cells, and monocytes than those of the low-risk expression subtype, and M0 macrophages were the higher one (Fig 6B). Spearman correlations of risk score and immune score were performed. The same result was shown in Fig 6C, that the risk score was negatively correlated with naïve B cells, resting memory CD4+ T cells, and monocytes (all p < 0.01). At the same time, there was a positive correlation between risk score and M0 macrophages(p<0.001). Then, ssGSEA analysis was carried out to reanalyze and examine the distinction between TICs and immune-related functions in two subgroups. Fig 6D demonstrates the dramatically different TIC abundance in the two categories, with 7/16 TICs showing the most significant variation, including 6 highly expressed types and just 1 downregulated type in the low-risk group. Several immune functional gene sets were enriched in the high-risk category for the C7 collection obtained from MSigDB’s immunologic gene set (Fig 6E). Based on the above analyses, B cells, CD4+ T cells, monocytes, M0 macrophages, and DCs were the most critical TICs in TCGA dataset HCC samples. Meanwhile, the immune activity of TME in the GSE14520 cohort was shown in S2 Fig. According to results from the two cohorts, memory CD4+ T cells, M0 macrophages, and Mast cells were the most correlated TICs between the hypoxia-related signature and TME (S4A-S4C Fig). The ssGEAG analysis complemented the immune activity of TME, which showed that the aDCs, macrophages, neutrophils, and Th2 cells infiltrating in the high-risk group were higher than in the low-risk group (S4D Fig). The immune functional gene sets of macrophages, DCs, neutrophils, and CD4+ T cells were enriched in the high-risk group (S4E Fig). In the LIHC_GSE140228_Smartseq2 dataset, 10 cell types were found, including B Cells, Conventional CD4 T Cells, Exhausted CD8 T Cells, Dendritic Cells, Innate Lymphoid Cells, Mast Cells, Monocytes or Macrophages, Natural Killer Cells, Plasma Cells and Proliferating T Cells (S5A Fig) The results further confirmed that FDPS was significantly highly expressed in Proliferating T Cells, SRM was significantly highly expressed in Proliferating T Cells and Innate Lymphoid Cells and NDRG1 was significantly highly expressed in Dendritic Cells and Monocytes or Macrophages (S5B Fig).

In conclusion, the analysis of immune infiltration in the GSE14520 and TCGA datasets mentioned above and scRNA-seq data indicated notable variations in immune cells in HCC patients, and there was a statistically significant link between the Hypoxia-Related Signature and TICs. Finally, we hypothesized that in HCC, CD4+ T cells, M0 macrophages, and DCs are most frequently associated with the Hypoxia-Related Signature. According to the scRNA-seq data, the 3 hypoxia-genes that constitute prognostic signature play different roles in the above TME cells.

### The predictive ability of the hypoxia-related signature for single-nucleotide, polymorphisms, chemotherapy drug sensitivity and immune checkpoints

**Fig 7:**
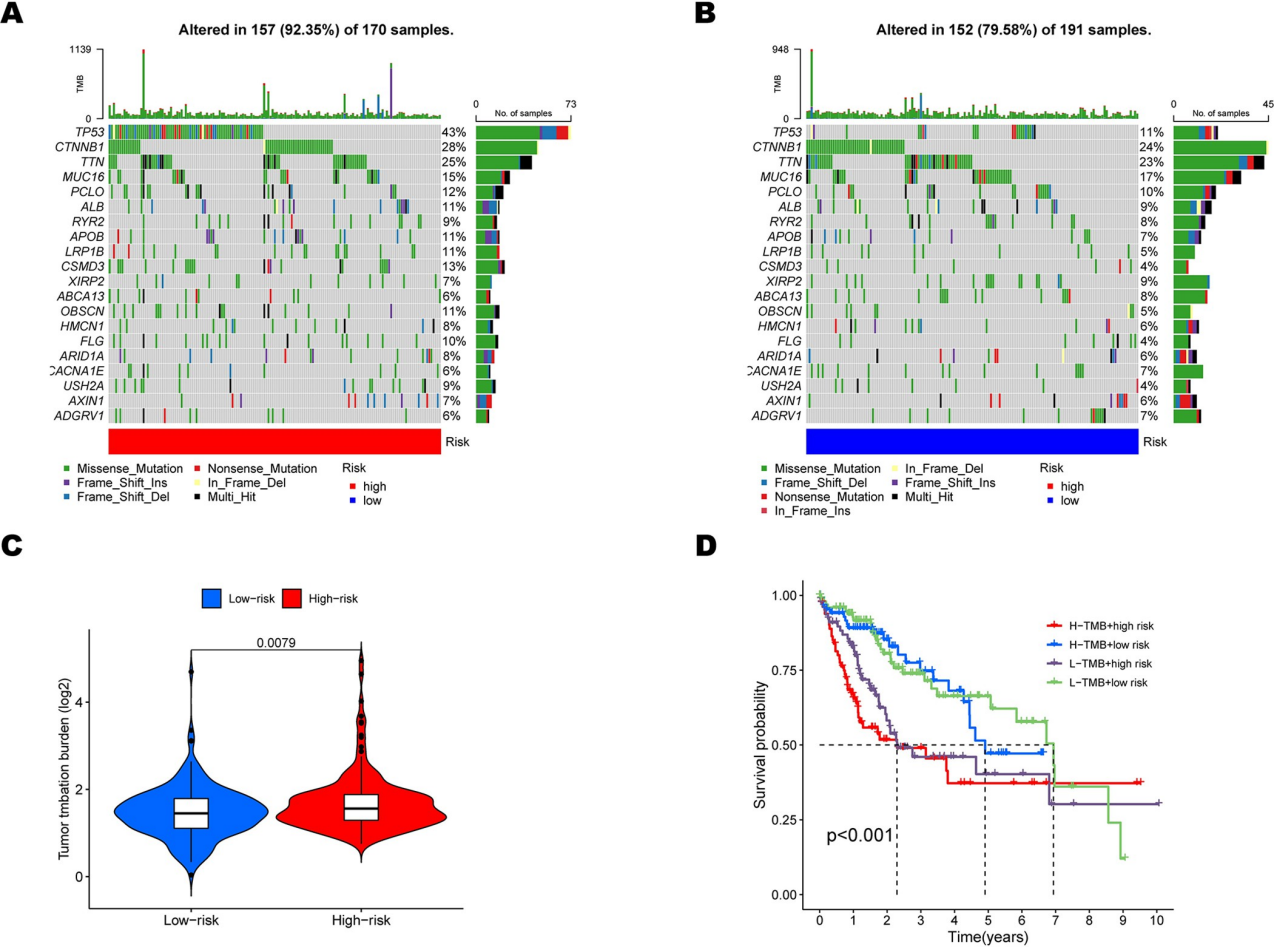
Characteristics of risk score with tumor somatic mutation and TMB in the TCGA dataset. (A) The waterfall plot of tumor somatic mutation in the high-risk subgroups(B)and the low-risk subgroups. (C) TMB distribution in the high and low-risk groups. (D) Kaplan–Meier survival curves for the HCC patients between high-TMB high-risk, high-TMB low-risk, low-TMB high-risk, and low-TMB low-risk subgroups.

**Fig 8:**
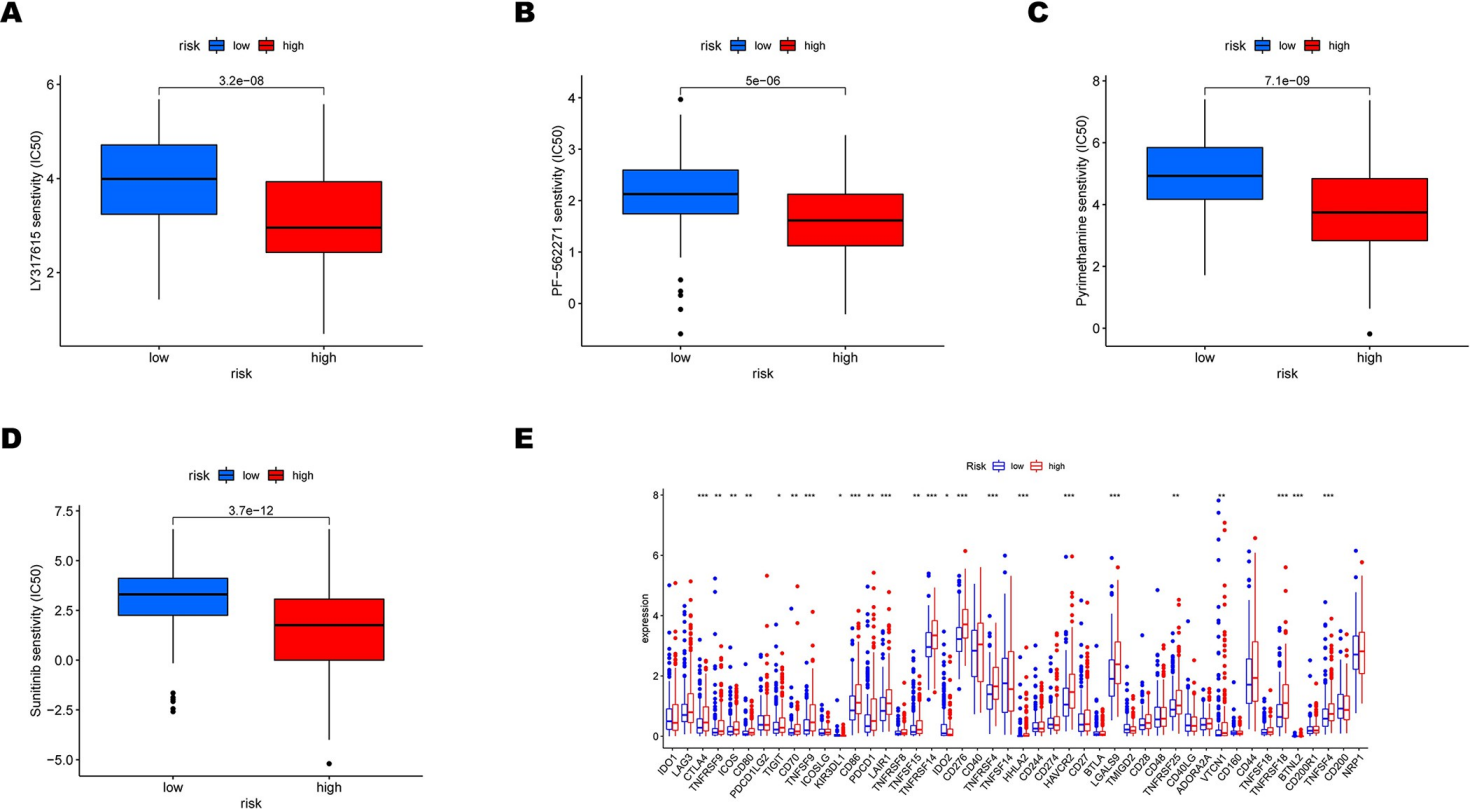
Association of risk score with chemotherapy sensitivity and the expression of immune checkpoints in the TCGA cohort. (A-D) Estimated IC50 for (A) LY317615, (B) PF−562271, (C) Pyrimethamine, and (D) Sunitinib in high and low-risk subgroups. (E) The expression level of possible immune checkpoints in high and low-risk groups.

It has been reported that affecting the epigenetic status in hepatocytes could play a role in HCC development [19]. Therefore, we compared the mutation landscapes between the high and low-risk patients in the TCGA cohort (Fig 7A and 7B). There was a higher mutation rate in 157 patients with high risk than in 152 patients in the low-risk subgroup (92.35% vs 79.58%). Regarding the gene mutation frequency, TP53, CTNNB1, and TTN were the most altered genes in high-risk patients than in low-risk patients (43% vs 11%, 28% vs 24%, and 25% vs 23%, respectively). Moreover, missense mutation was the most common mutation type in HCC patients. Meanwhile, Tumor mutation burden(TMB) was also evaluated in our study. TMB in the high-risk subgroup was more extensive than in the low-risk subgroup (p<0.01, Fig 7C). At the same time, there was significant difference in Kaplan–Meier survival analysis between the high or low TMB sorted by the median and high or low risk subgroups (p<0.001, Fig 7D). The TCGA and GSE14520 dataset examined the connection between risk score and clinical chemotherapeutic drug sensitivity. LY317615, PF562271, Pyrimethamine, and Sunitinib patients exhibited lower estimated IC50s in the low-risk group, as shown in Fig 8A–8D and S6A–S6D Fig (all p<0.001), indicating that HCC with high risk may benefit from these chemotherapeutic drugs. Finally, how immune checkpoint expression varied across high and low-risk groups was investigated. Increased expression of CD86, LAIR1, and LGALS9 in the high-risk group raises the possibility that these individuals will respond to immunotherapy more favorably. (all p<0.05, Fig 8E and S6E Fig).

## Discussion

Hepatocellular carcinoma (HCC) is one of the cancers with high mortality in the world, which arises from hepatocytes through the sequential accumulation of multiple genomic and epigenomic alterations [20]. Although HCC has traditionally had limited therapeutic options, such as surgical resection, liver transplantation, and bridge therapy, it finally turns out to be a poor prognosis [21]. It has been known that hypoxia promotes HCC invasion and metastasis [22], and there was a marked correlation between the hypoxic microenvironment and sorafenib resistance [23] and progression [24] in HCC. To identify the role of the hypoxic microenvironment in the progression of HCC and enhance treatment outcomes, novel therapeutic approaches for HCC must be developed. To do this, high-throughput sequencing and data analysis are required to identify novel hypoxic-related prognostic biomarkers [25]. Based on the TCGA and GSE14520 dataset, we identified 25 commonly differentially expressed hypoxia-related genes in this work using GSEA and WCGNA. Using the LASSO Cox regression analysis, a 3-gene hypoxia risk signature was created. FDPS was confirmed to promote prostate cancer progression and glioma growth through modulation of small GTPases/AKT axis [26] or regulating CCL20 via the Wnt/β-catenin signaling pathway [27]. For SRM, it has been reported that SRM was one member of the signature that showed good performance in predicting carcinogenesis and bad OS in HCC [28]. Previous studies have shown that NDRG1 was an adverse prognostic indicator for metastasis, recurrence, and poor prognosis in HCC [29]. Afterward, it was found that NDRG1 exacerbates HCC initiation via enhancing crosstalk between fibroblasts and tumor cells [30] and promotes the growth of HCC cells by interacting with GSK-3β and Nur77 to prevent β-catenin degradation [31]. Consequently, these 3 HGs play a vital part during HCC tumorization and could be clinically valuable as potential biomarkers.

According to the clinical correlation study, there is a strong link between the risk score and tumor grade, CLIP stage, BCLC stage, TNM stage, and pathological T stage. Notably, the 3-gene hypoxia risk signature significantly impacted prognosis estimates for different HCC subtypes when they were categorized by age, gender, and clinical TNM stage in stratified survival analysis. In both the training, testing, and validation cohorts, the 1-, 3-, and 5-year AUC values of the ROC curves for the 3-gene signature were higher than 0.60. The calibration curves for the 1-, 3-, and 5-year nomogram predictions demonstrated good agreement between the predicted values and the actual measurements. The risk signature of the three HGs may be identified as independent prognostic variables for predicting the survival of HCC patients after univariate and multivariate Cox regression studies.

HCC is distinguished by a great number of infiltrated immune cells, suppressive molecules, and intricate interactions between different components. The tumor immune microenvironment in HCC plays a crucial role in HCC progression and recurrence [32]. Previous studies showed that activated CD4+ T cells from HCC interact with macrophages in laboratory mice, leading to cytokines produced by macrophages that reduce the anti-tumor immune response [33]. It has been identified that high infiltration levels of M0 macrophage [34] and DC [35] were more likely to have bad OS than patients with low M0 macrophage or DC expression in HCC. Immune tolerance in hepatocellular cancer may be related to DCs-elicited B-cell activation [36]. When taken as a whole, patients of the high-risk subgroup contained higher infiltration levels of activated CD4+ T cells, M0 macrophages, and DCs while having lower infiltration levels of resting CD4+ T cells than those of the low-risk subgroup.

In this study, we went further into the HGs signature mutation landscape. Somatic mutation count was higher in the high-risk subgroup. In particular, it was shown that patients in the high-risk subgroup had TP53 mutations at much greater rates (43% vs. 11%). The TP53 gene is mutated in more than 50% of human cancers, and this mutation promotes tumorigenesis and tumor development but not HCC [37]. Hypoxia inducible factor-1α (HIF1α) is a master driver of stemness in HCC cells under hypoxic conditions of which TP53 are direct targets [38]. In hypoxia-induced HCC cells, DUSP18 finally inhibits MAPK14-mediated TP53 phosphorylation and the stability of TP53 protein, which ultimately promotes HCC cell migration, invasion, and cell cycle [39]. As a result, the increased incidence of TP53 mutation seen in HCC patients in the high-risk category may have an effect on the subgroup’s hypoxic phenotype. The hypoxic tumor microenvironment also affects the metabolism of tumor tissue, in which hypoxic tumor cells have a clear preference for glutamine metabolism. Under hypoxic conditions, tumor cells increase glutamine uptake [40] and activate additional metabolic pathways required for tumor growth [40, 41]. However, the immunosuppressive microenvironment of the tumor is reversed by glutamine antagonists, resulting in reduced hypoxia, acidosis, and nutritional depletion. Effector T cells are activated in this circumstance, resulting in a powerful anti-tumor response [42].

During the treatment of HCC, some patients can be treated with chemotherapy, where chemotherapy resistance and metastasis act as major obstacles. The development of new chemotherapy agents and the identification of chemosensitive individuals may enhance the effectiveness of the anti-tumor course in HCC patients. Previous studies have shown that HCC patients could benefit from LY317615 [43], PF−562271 [44], Pyrimethamine [45], and Sunitinib [46]. Patients in the high-risk subgroup responded more sensitively to the chemotherapy mentioned above, which was administered in accordance with the GDSC database. This discovery adds further knowledge to the drug selection process. However, additional clinical studies are necessary to determine the precise efficacy of the latent medicines throughout the treatment of HCC. In the immune checkpoint differential expression examination, CD86, LAIR1, and LGALS9 were highly expressed in the high-risk subgroup, indicating the need for novel immunotherapy targeting these immune checkpoints.

Certain limitations of this study should be noted. Firstly, although relatively loose filtering conditions were conducted in the present work for independent external validation, several HGs were eliminated in standardization and adjusting the weight of the regression coefficient in LASSO. Second, as all data were gathered from public databases retroactively, it’s possible that not all changes in HCC cases from other geographic regions at different times are included. Moreover, our research was based on molecular biology, and our findings about immune infiltration, drug sensitivity, and immunotherapy need to be further validated in HCC case research.

## Conclusions

To sum up, after hypoxia-related pathways were defined in HCC, to stratify HCC patients and predict OS for HCC, a prognostic signature made up of three HGs is created and validated. Patients in the high-risk subgroups had lower survival rates, higher levels of TICs infiltration, higher levels of mutations, and a variety of potential treatments, which provides a comprehensive perspective for clinicians to decide the diagnosis and treatment of HCC.

## Supporting information

S1 Fig Validation of prognostic risk signature of HGs for HCC patients in LIRI-JP cohort. (A-B) Survival status of patients, risk plot distribution, and (C) heatmap of expression of 3 core HGs. (D) Kaplan–Meier survival curves for the risk signature based on the training, testing and validation cohort. (E) Receiver operating characteristic (ROC) curves for the risk signature in the training, testing and validation cohort.
(TIF)

S2 Fig Identification of the risk signature with the overall survival and clinicopathological characteristics of HCC patients ground on the GSE14520 dataset. (A) Relationships between the risk score and clinicopathological characteristics of HCC patients. (B) Forest maps of the univariate and multivariate Cox regression analysis between the risk score and clinical characteristics. (C) Nomogram predicting the survival rate at 1, 3, 5 years for HCC patients, *p < 0.05, **p < 0.01, and ***p < 0.001. (D) Calibration plots for the nomogram.
(TIF)

S3 Fig Identification of the risk signature with the overall survival and clinicopathological characteristics of HCC patients ground on the LIRI-JP cohort. (A) Relationships between the risk score and clinicopathological characteristics of HCC patients. (B) Forest maps of the univariate and multivariate Cox regression analysis between the risk score and clinical characteristics. (C) Nomogram predicting the survival rate at 1, 3, 5 years for HCC patients, *p < 0.05, **p < 0.01, and ***p < 0.001. (D) Calibration plots for the nomogram.
(TIF)

S4 Fig Association of tumor immune cell infiltration with the risk score in the GSE14520 cohort. (A) The bar graph of relative proportions of the 22 immune cells between the high and low-risk subgroups. (B) The bar graph of difference in composition of the 22 types of immune cells between two risk subgroups, *p < 0.05, **p < 0.01, and ***p < 0.001. (C) The correlation between the risk score and resting memory CD4 T cells, activated memory CD4 T cells, M0 macrophages, resting mast cells, and activated mast cells. (D) The bar graph of the difference in the enrichment scores of 16 types of immune cells between two risk subgroups. (E) The multiple GSEA for significant immune pathways based on the GSE14520 datasets.
(TIF)

S5 Fig Single-cell analysis identified the expression of 3 hypoxia-related genes. (A) Umap of 10 cell types in GSE140228. (B) A heatmap was be displayed to show average expression levels of 3 hypoxia-related genes.
(TIF)

S6 Fig Association of risk score with chemotherapy sensitivity and the expression of immune checkpoints in the GSE14520 cohort. (A-D) Estimated IC50 for (A) LY317615, (B) PF−562271, (C) Pyrimethamine, and (D) Sunitinib in high and low-risk subgroups. (E) The expression level of possible immune checkpoints in high and low-risk groups.
(TIF)
