# Supplementary figures and images for: Construction and validation of 3-genes hypoxia-related prognostic signature to predict the prognosis and therapeutic response of hepatocellular carcinoma patients
Source: PLoS One. 2023 Jul 5;18(7):e0288013. doi: 10.1371/journal.pone.0288013 (PMC10321610; doi:10.1371/journal.pone.0288013)

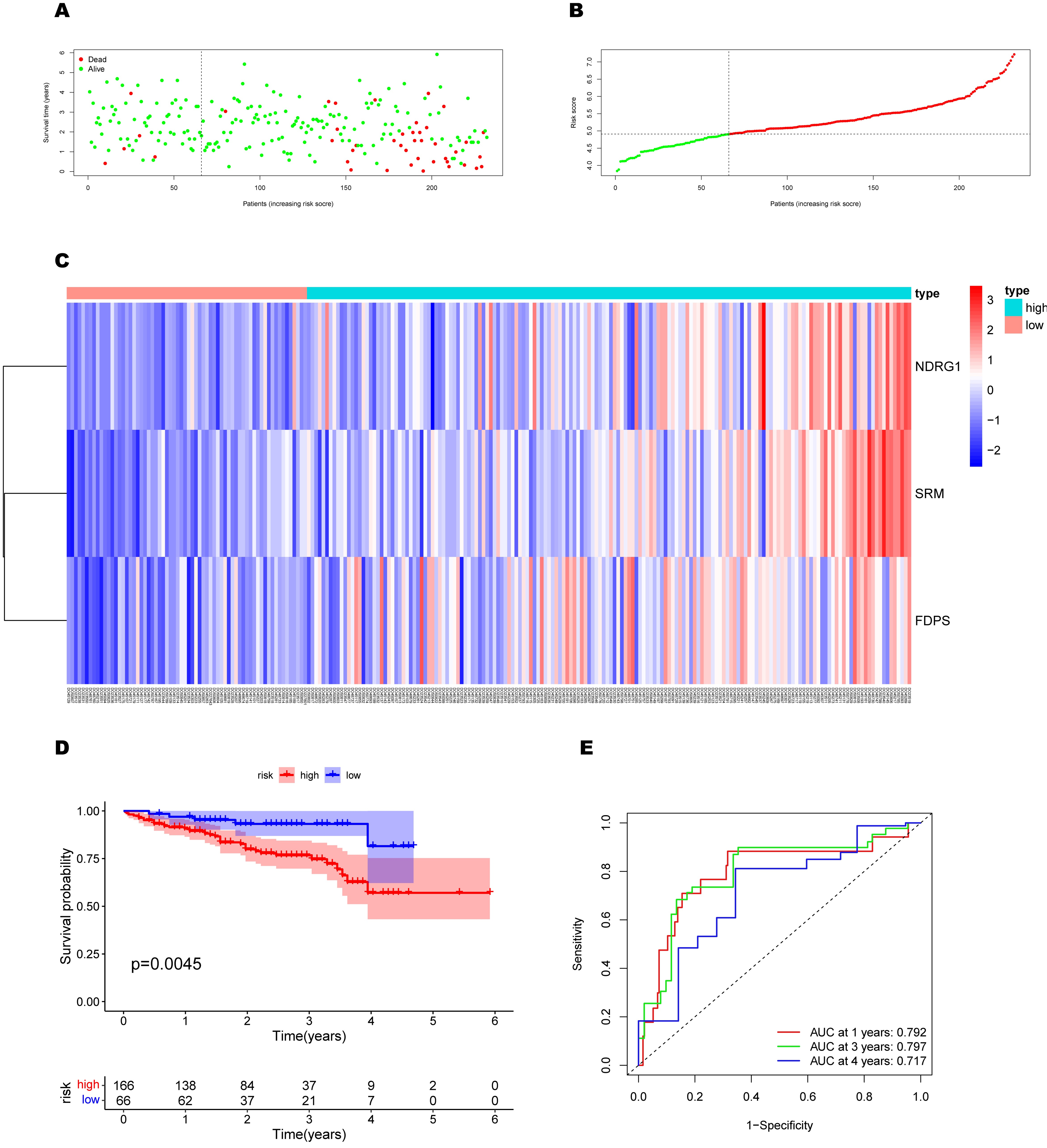

Supplement: S1 Fig — (A-B) Survival status of patients, risk plot distribution, and (C) heatmap of expression of 3 core HGs. (D) Kaplan–Meier survival curves for the risk signature based on the training, testing and validation cohort. (E) Receiver operating characteristic (ROC) curves for the risk signature in the training, testing and validation cohort. (TIF) [file pone.0288013.s001.tif]

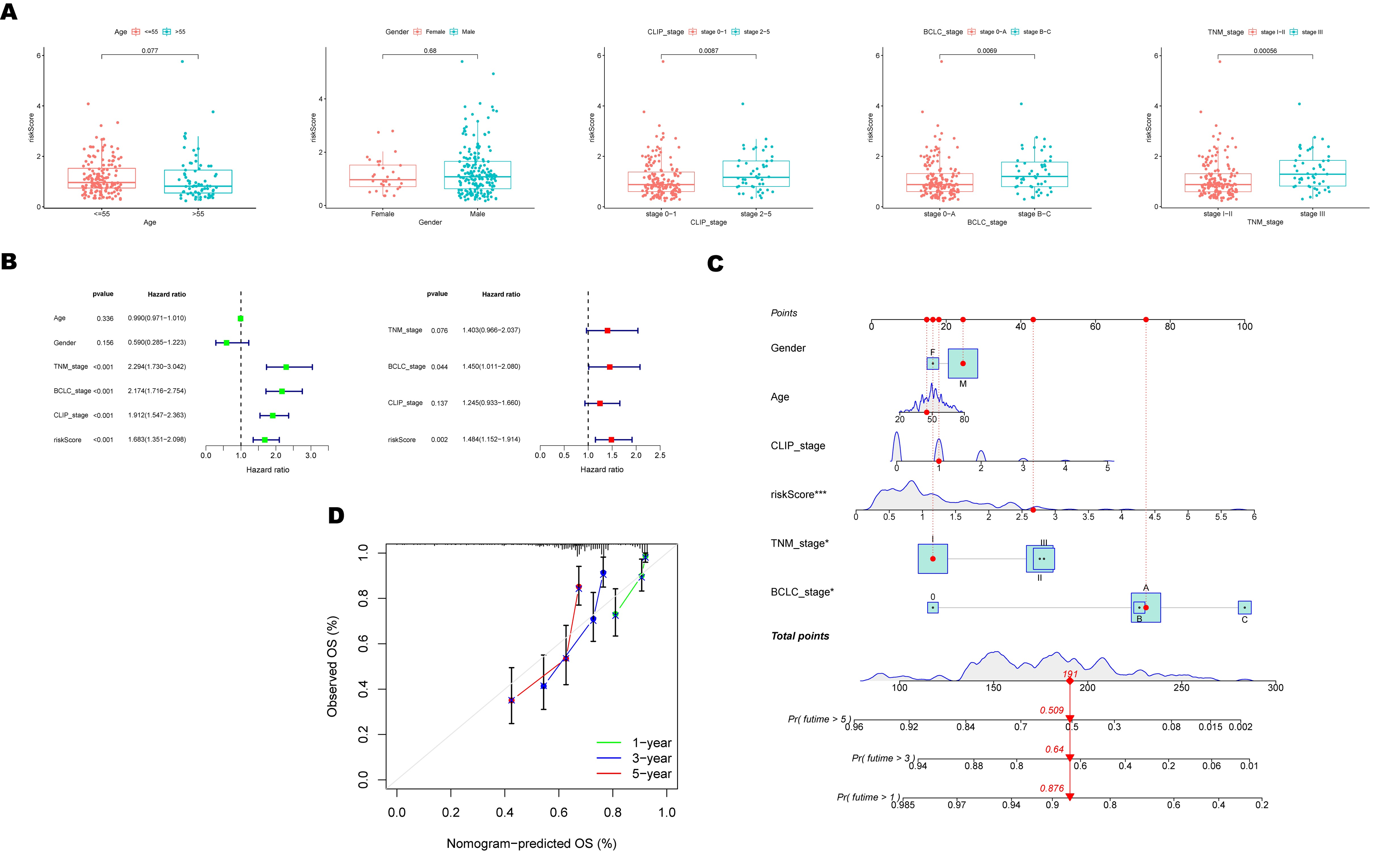

Supplement: S2 Fig — (A) Relationships between the risk score and clinicopathological characteristics of HCC patients. (B) Forest maps of the univariate and multivariate Cox regression analysis between the risk score and clinical characteristics. (C) Nomogram predicting the survival rate at 1, 3, 5 years for HCC patients, *p < 0.05, **p < 0.01, and ***p < 0.001. (D) Calibration plots for the nomogram. (TIF) [file pone.0288013.s002.tif]

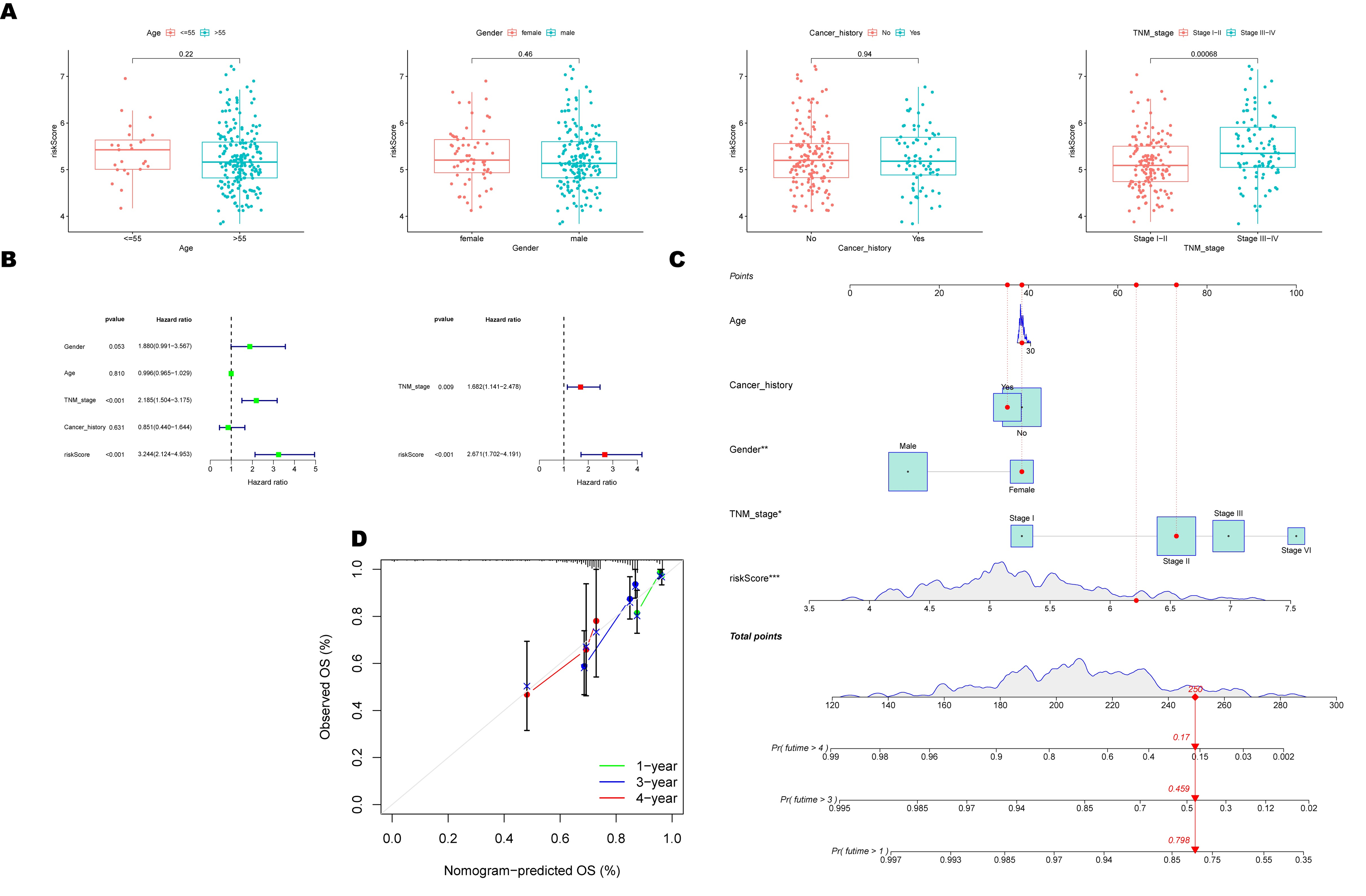

Supplement: S3 Fig — (A) Relationships between the risk score and clinicopathological characteristics of HCC patients. (B) Forest maps of the univariate and multivariate Cox regression analysis between the risk score and clinical characteristics. (C) Nomogram predicting the survival rate at 1, 3, 5 years for HCC patients, *p < 0.05, **p < 0.01, and ***p < 0.001. (D) Calibration plots for the nomogram. (TIF) [file pone.0288013.s003.tif]

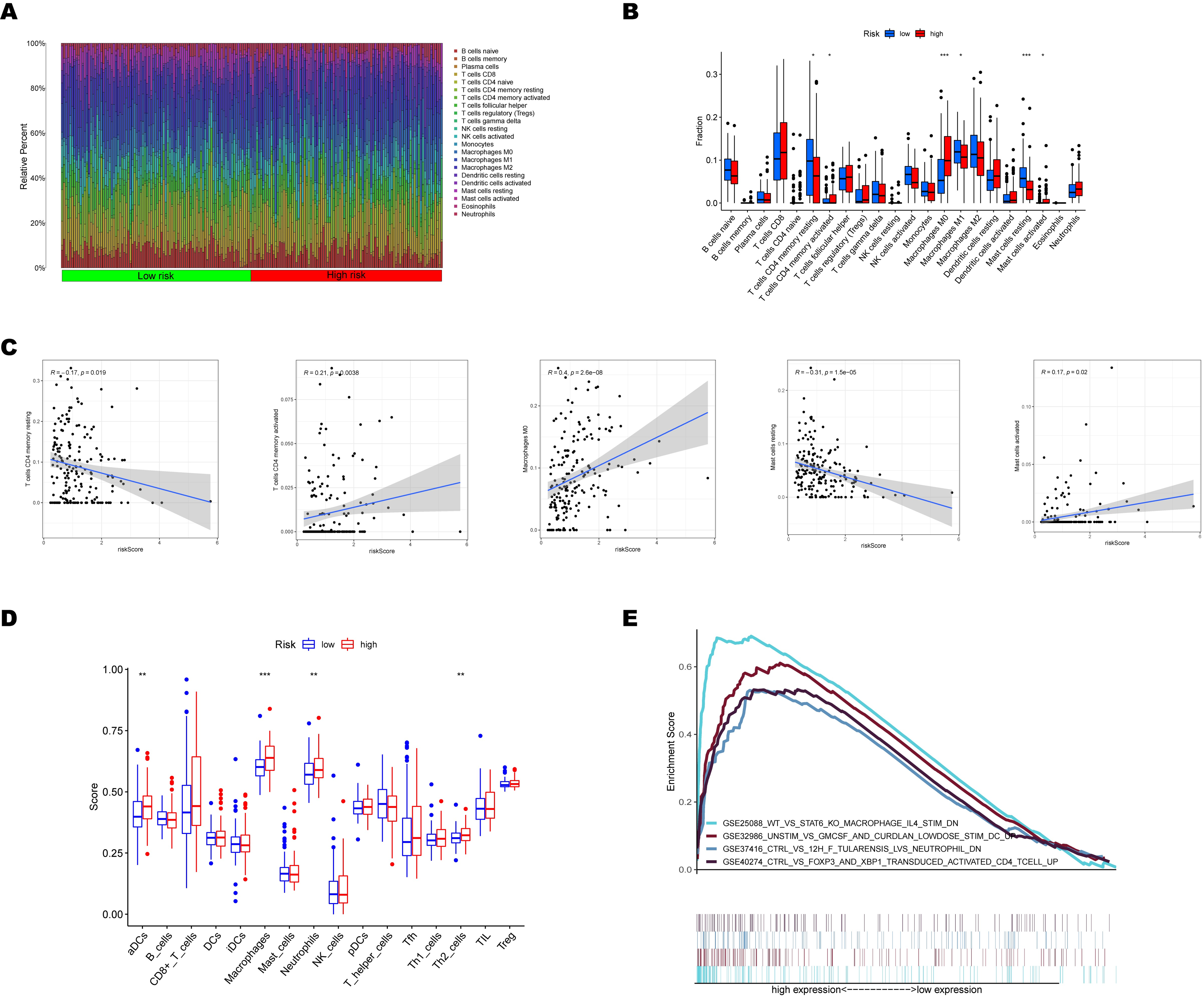

Supplement: S4 Fig — (A) The bar graph of relative proportions of the 22 immune cells between the high and low-risk subgroups. (B) The bar graph of difference in composition of the 22 types of immune cells between two risk subgroups, *p < 0.05, **p < 0.01, and ***p < 0.001. (C) The correlation between the risk score and resting memory CD4 T cells, activated memory CD4 T cells, M0 macrophages, resting mast cells, and activated mast cells. (D) The bar graph of the difference in the enrichment scores of 16 types of immune cells between two risk subgroups. (E) The multiple GSEA for significant immune pathways based on the GSE14520 datasets. (TIF) [file pone.0288013.s004.tif]

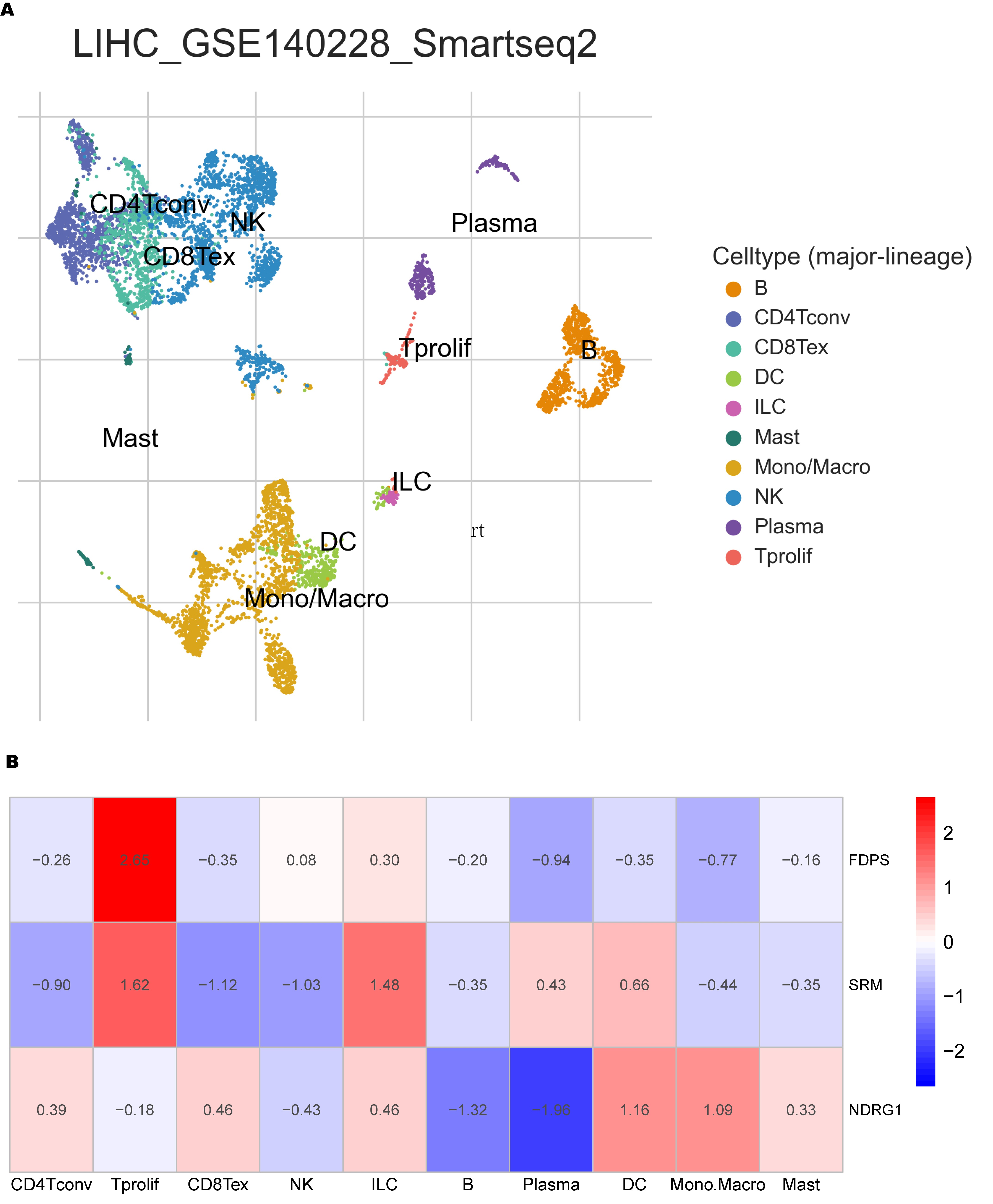

Supplement: S5 Fig — (A) Umap of 10 cell types in GSE140228. (B) A heatmap was be displayed to show average expression levels of 3 hypoxia-related genes. (TIF) [file pone.0288013.s005.tif]

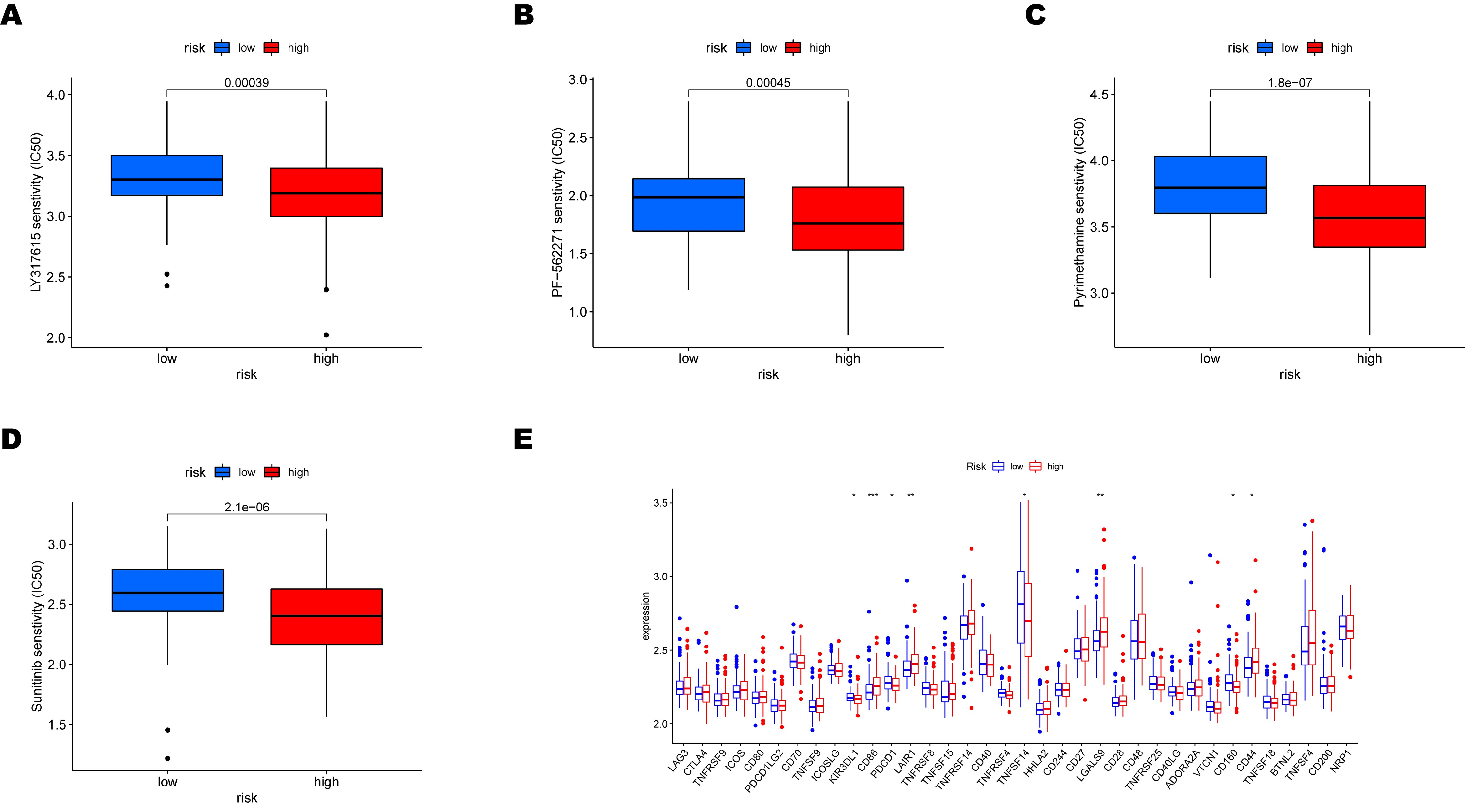

Supplement: S6 Fig — (A-D) Estimated IC50 for (A) LY317615, (B) PF−562271, (C) Pyrimethamine, and (D) Sunitinib in high and low-risk subgroups. (E) The expression level of possible immune checkpoints in high and low-risk groups. (TIF) [file pone.0288013.s006.tif]
